# Supplementary material for: Mitochondrial Genome of Fagopyrum esculentum and the Genetic Diversity of Extranuclear Genomes in Buckwheat
Source: Plants (Basel). 2020 May 12;9(5):618. doi: 10.3390/plants9050618 (PMC7285332; doi:10.3390/plants9050618)
Supplement: Supplementary file 1 [file plants-09-00618-s001.zip › Figure_S2.pdf]

Supplementary figure S2. Phylogenetic trees based on ML analysis of single mitochondrial gene sequences. Bootstrap support values above 50 are shown; nodes with lower support are collapsed.

atp1

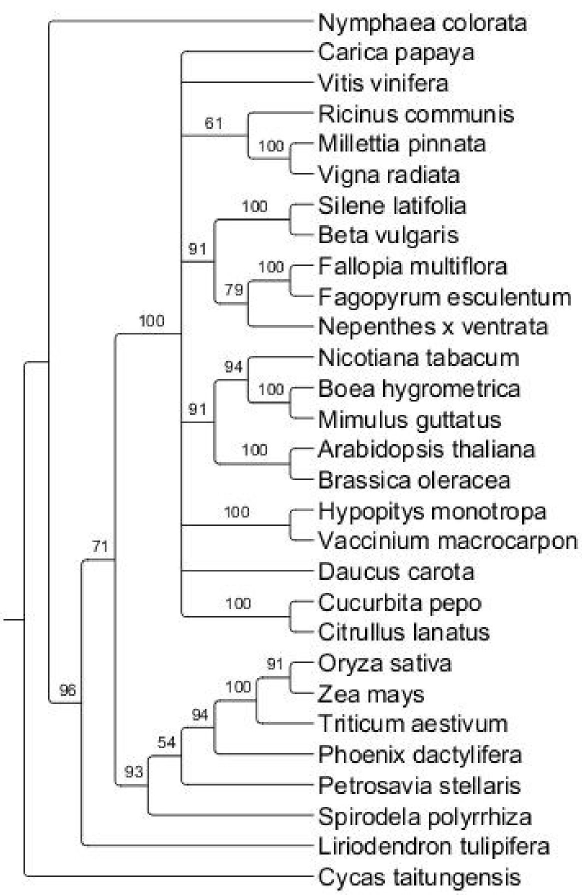

atp4

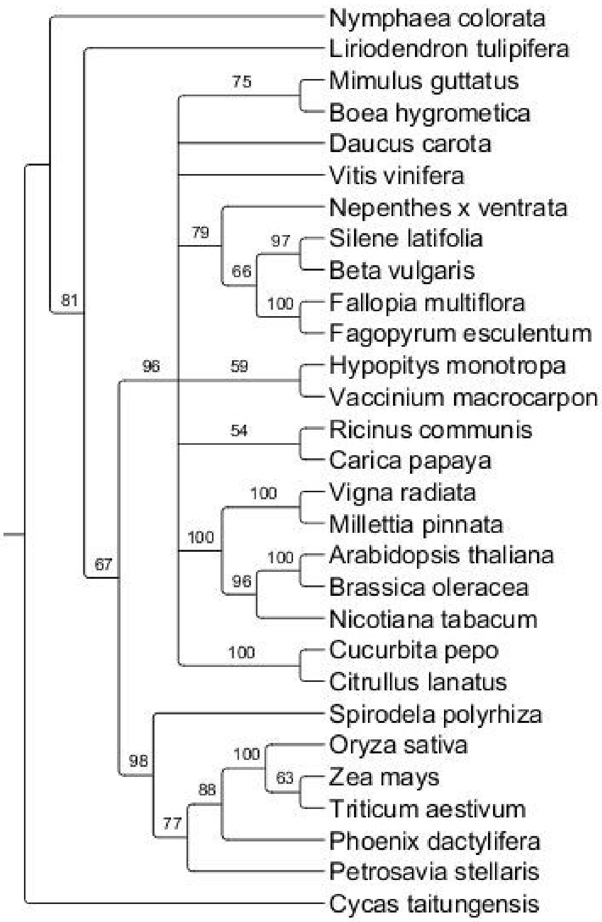

atp8

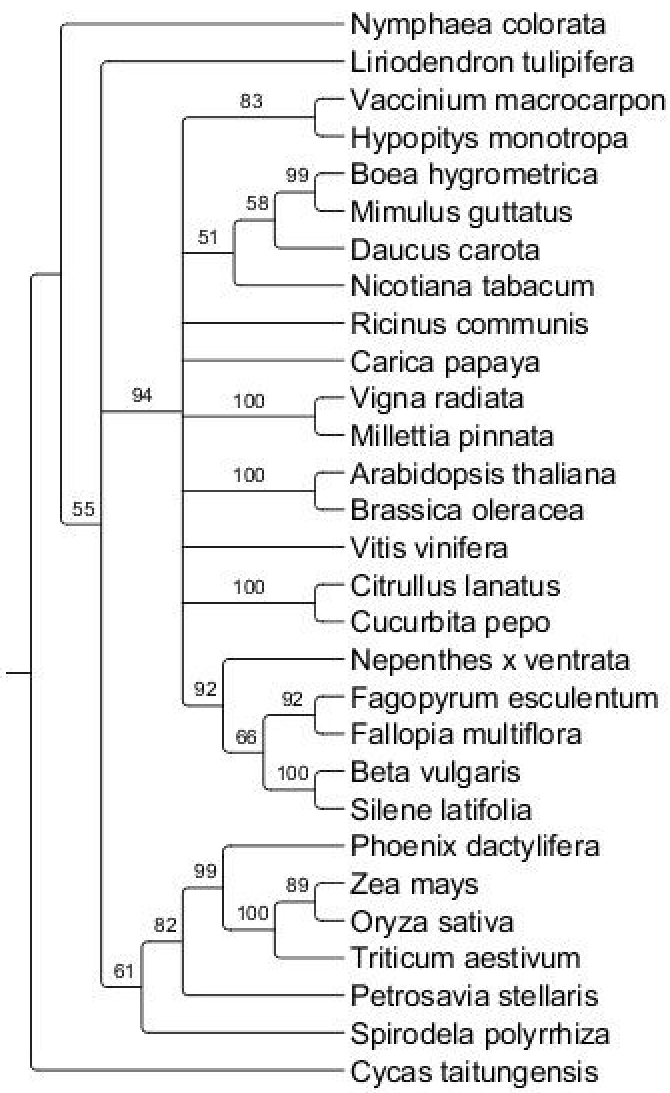

Nymphaea colorata
Liriodendron tulipifera
83
Vaccinium macrocarpon
Hypopitys monotropa
99
Boea hygrometrica
58
Mimulus guttatus
51
Daucus carota
Nicotiana tabacum
Ricinus communis
Carica papaya
94
100
Vigna radiata
Millettia pinnata
100
Arabidopsis thaliana
55
Brassica oleracea
Vitis vinifera
100
Citrullus lanatus
Cucurbita pepo
Nepenthes x ventrata
92
92
Fagopyrum esculentum
66
Fallopia multiflora
100
Beta vulgaris
Silene latifolia
Phoenix dactylifera
99
89
Zea mays
82
100
Oryza sativa
Triticum aestivum
61
Petrosavia stellaris
Spirodela polyrrhiza
Cycas taitungensis

atp9

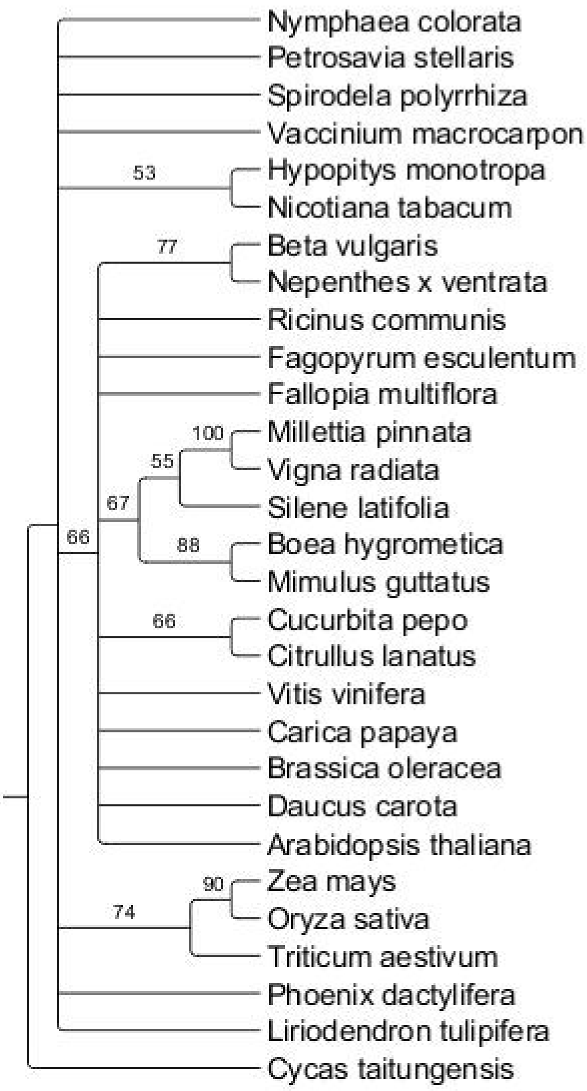

Nymphaea colorata
Petrosavia stellaris
Spirodela polyrrhiza
Vaccinium macrocarpon
53
Hypopitys monotropa
Nicotiana tabacum
77
Beta vulgaris
Nepenthes x ventrata
Ricinus communis
Fagopyrum esculentum
Fallopia multiflora
100
Millettia pinnata
55
Vigna radiata
67
Silene latifolia
66
88
Boea hygrometica
Mimulus guttatus
66
Cucurbita pepo
Citrullus lanatus
Vitis vinifera
Carica papaya
Brassica oleracea
Daucus carota
Arabidopsis thaliana
90
Zea mays
74
Oryza sativa
Triticum aestivum
Phoenix dactylifera
Liriodendron tulipifera
Cycas taitungensis

ccmC

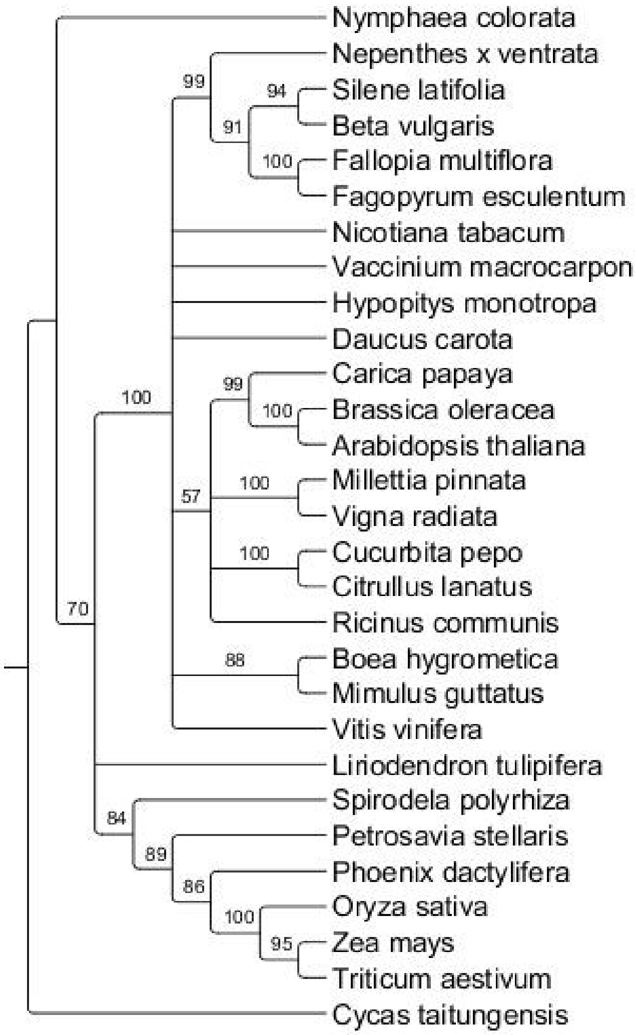

Nymphaea colorata
Nepenthes x ventrata
Silene latifolia
Beta vulgaris
Fallopia multiflora
Fagopyrum esculentum
Nicotiana tabacum
Vaccinium macrocarpon
Hypopitys monotropa
Daucus carota
Carica papaya
Brassica oleracea
Arabidopsis thaliana
Millettia pinnata
Vigna radiata
Cucurbita pepo
Citrullus lanatus
Ricinus communis
Boea hygrometica
Mimulus guttatus
Vitis vinifera
Liriodendron tulipifera
Spirodela polyrhiza
Petrosavia stellaris
Phoenix dactylifera
Oryza sativa
Zea mays
Triticum aestivum
Cycas taitungensis
99
94
91
100
100
99
100
57
100
100
88
70
84
89
86
100
95

cob

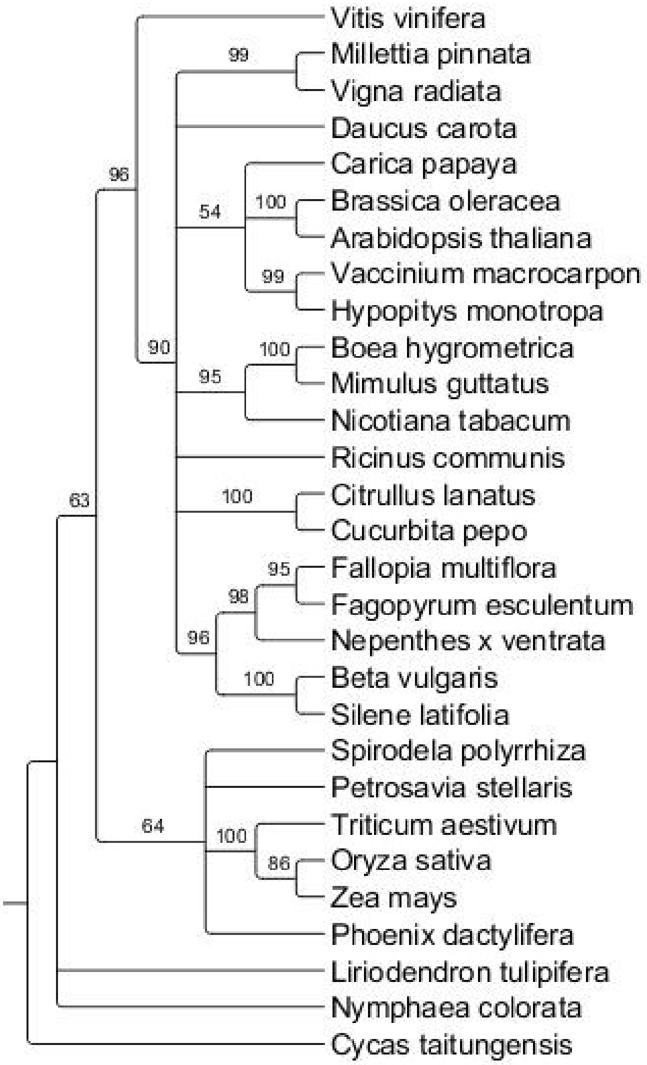

Vitis vinifera
Millettia pinnata
Vigna radiata
Daucus carota
Carica papaya
Brassica oleracea
Arabidopsis thaliana
Vaccinium macrocarpon
Hypopitys monotropa
Boea hygrometrica
Mimulus guttatus
Nicotiana tabacum
Ricinus communis
Citrullus lanatus
Cucurbita pepo
Fallopia multiflora
Fagopyrum esculentum
Nepenthes x ventrata
Beta vulgaris
Silene latifolia
Spirodela polyrrhiza
Petrosavia stellaris
Triticum aestivum
Oryza sativa
Zea mays
Phoenix dactylifera
Liriodendron tulipifera
Nymphaea colorata
Cycas taitungensis
99
96
54
100
99
90
100
95
100
63
95
98
96
100
64
100
86

cox1

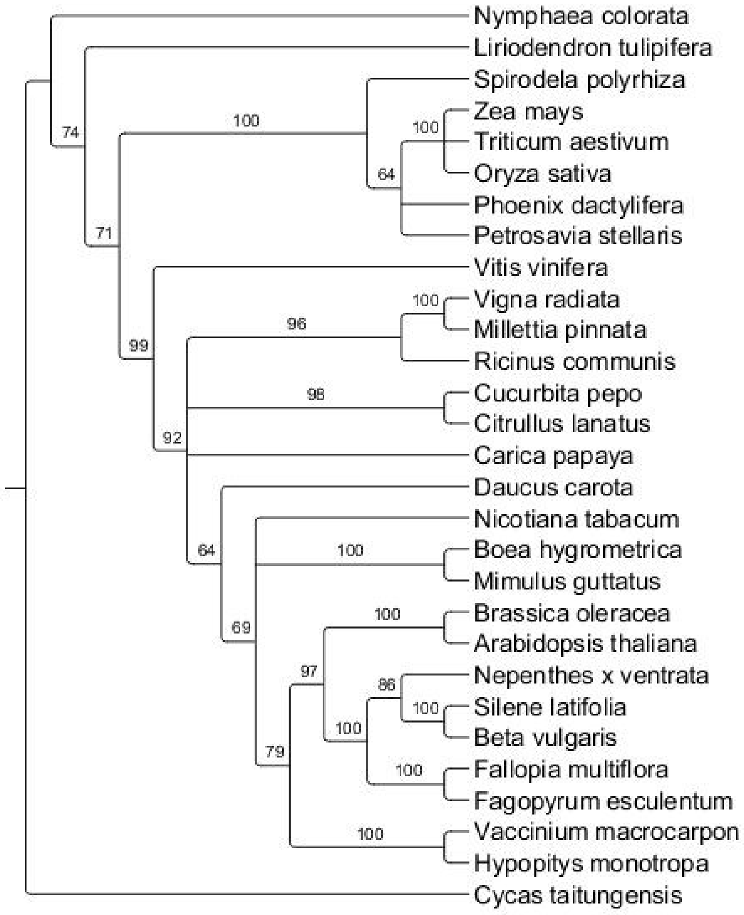
Nymphaea colorata
Liriodendron tulipifera
Spirodela polyrhiza
Zea mays
Triticum aestivum
Oryza sativa
Phoenix dactylifera
Petrosavia stellaris
Vitis vinifera
Vigna radiata
Millettia pinnata
Ricinus communis
Cucurbita pepo
Citrullus lanatus
Carica papaya
Daucus carota
Nicotiana tabacum
Boea hygrometrica
Mimulus guttatus
Brassica oleracea
Arabidopsis thaliana
Nepenthes x ventrata
Silene latifolia
Beta vulgaris
Fallopia multiflora
Fagopyrum esculentum
Vaccinium macrocarpon
Hypopitys monotropa
Cycas taitungensis

cox3

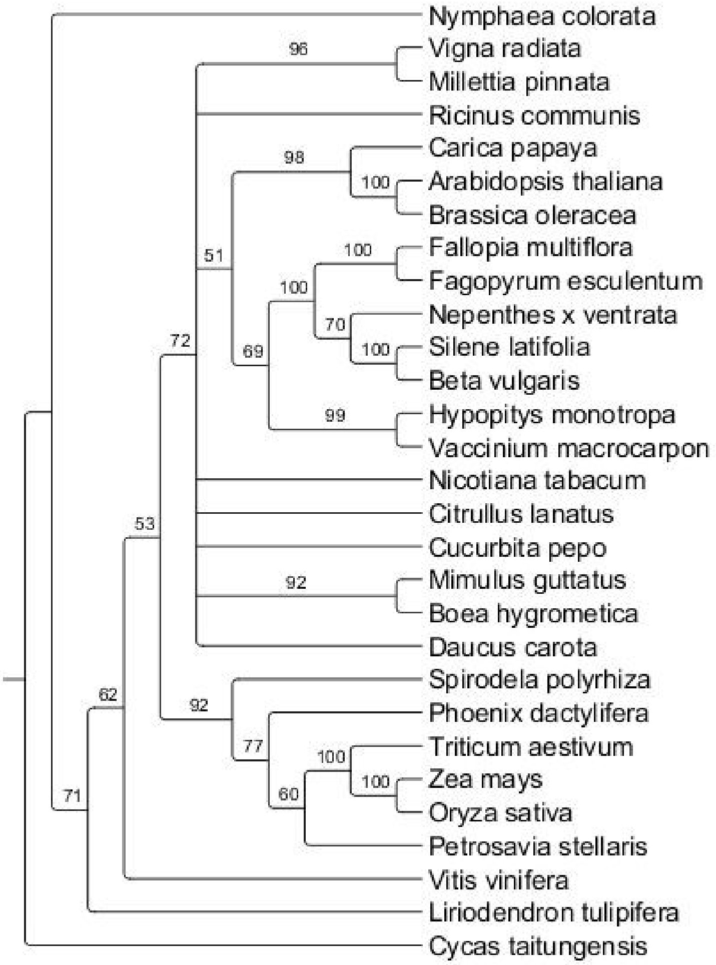
Nymphaea colorata
Vigna radiata
Millettia pinnata
Ricinus communis
Carica papaya
Arabidopsis thaliana
Brassica oleracea
Fallopia multiflora
Fagopyrum esculentum
Nepenthes x ventrata
Silene latifolia
Beta vulgaris
Hypopitys monotropa
Vaccinium macrocarpon
Nicotiana tabacum
Citrullus lanatus
Cucurbita pepo
Mimulus guttatus
Boea hygrometica
Daucus carota
Spirodela polyrhiza
Phoenix dactylifera
Triticum aestivum
Zea mays
Oryza sativa
Petrosavia stellaris
Vitis vinifera
Liriodendron tulipifera
Cycas taitungensis

matR

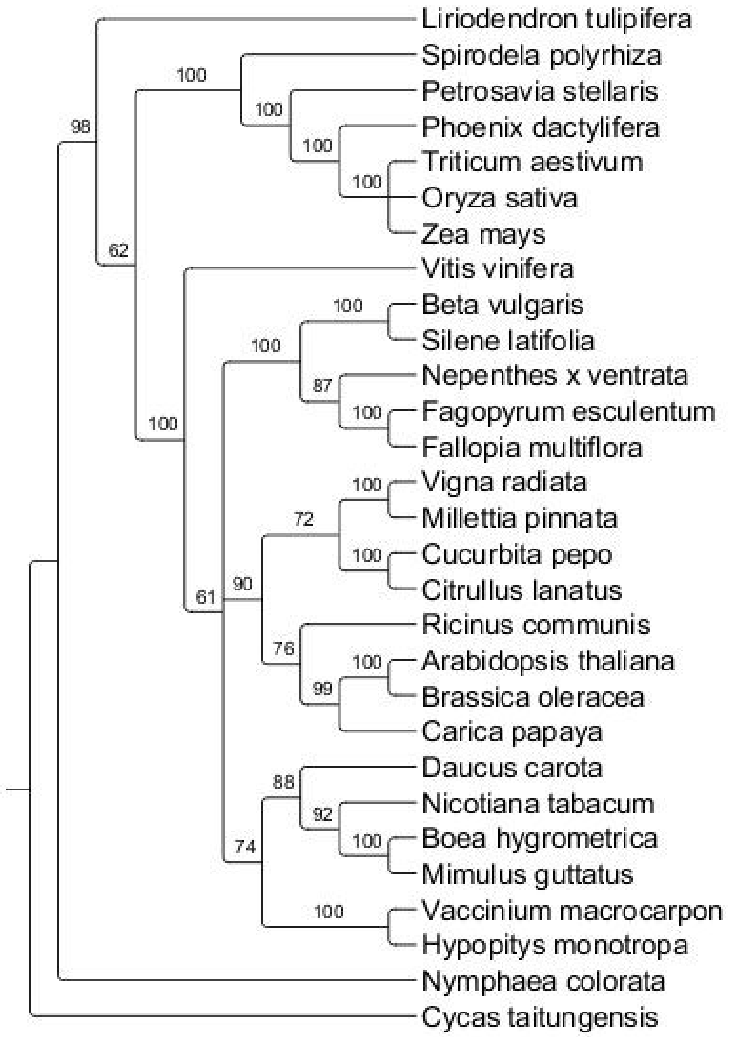

Liriodendron tulipifera
Spirodela polyrhiza
Petrosavia stellaris
Phoenix dactylifera
Triticum aestivum
Oryza sativa
Zea mays
Vitis vinifera
Beta vulgaris
Silene latifolia
Nepenthes x ventrata
Fagopyrum esculentum
Fallopia multiflora
Vigna radiata
Millettia pinnata
Cucurbita pepo
Citrullus lanatus
Ricinus communis
Arabidopsis thaliana
Brassica oleracea
Carica papaya
Daucus carota
Nicotiana tabacum
Boea hygrometica
Mimulus guttatus
Vaccinium macrocarpon
Hypopitys monotropa
Nymphaea colorata
Cycas taitungensis

nad1

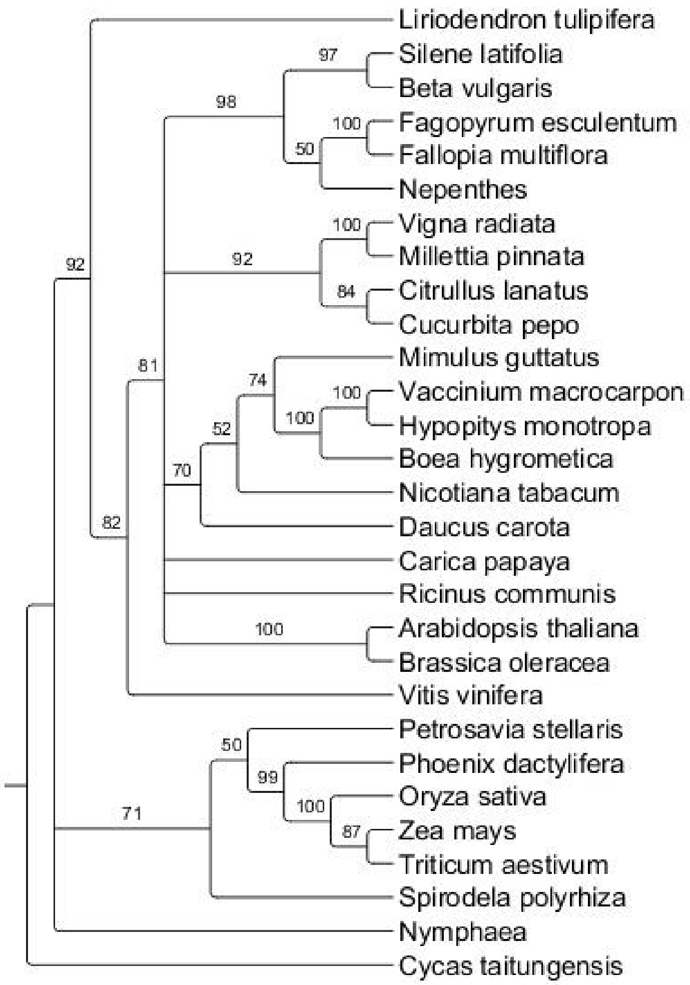

Liriodendron tulipifera
Silene latifolia
Beta vulgaris
Fagopyrum esculentum
Fallopia multiflora
Nepenthes
Vigna radiata
Millettia pinnata
Citrullus lanatus
Cucurbita pepo
Mimulus guttatus
Vaccinium macrocarpon
Hypopitys monotropa
Boea hygrometica
Nicotiana tabacum
Daucus carota
Carica papaya
Ricinus communis
Arabidopsis thaliana
Brassica oleracea
Vitis vinifera
Petrosavia stellaris
Phoenix dactylifera
Oryza sativa
Zea mays
Triticum aestivum
Spirodela polyrhiza
Nymphaea
Cycas taitungensis

nad2

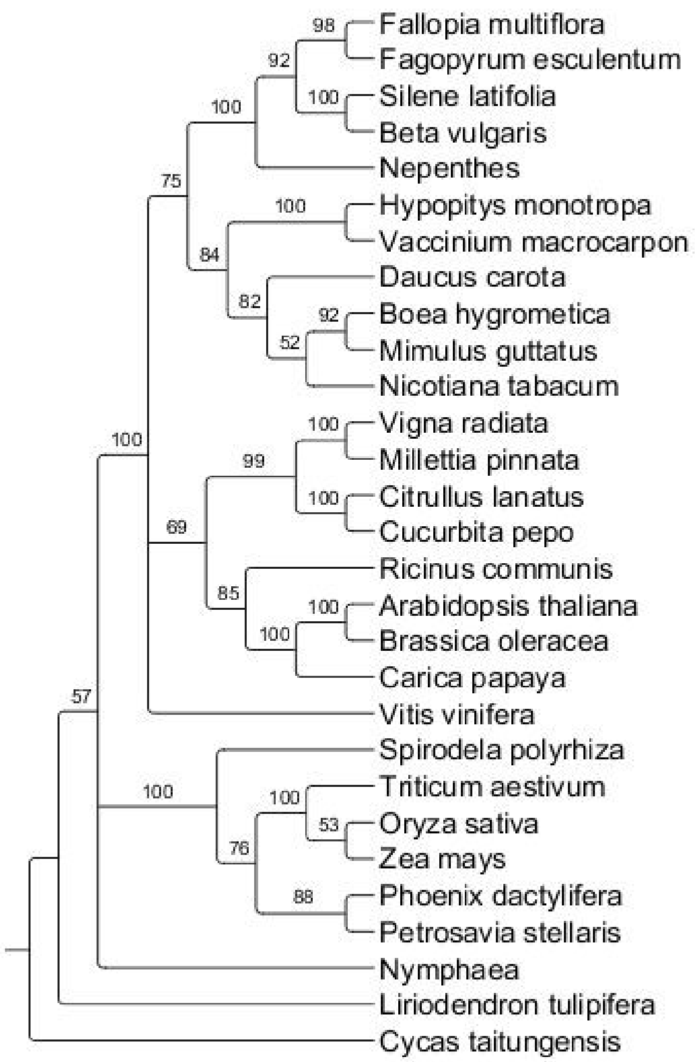
Fallopia multiflora
Fagopyrum esculentum
Silene latifolia
Beta vulgaris
Nepenthes
Hypopitys monotropa
Vaccinium macrocarpon
Daucus carota
Boea hygrometica
Mimulus guttatus
Nicotiana tabacum
Vigna radiata
Millettia pinnata
Citrullus lanatus
Cucurbita pepo
Ricinus communis
Arabidopsis thaliana
Brassica oleracea
Carica papaya
Vitis vinifera
Spirodela polyrhiza
Triticum aestivum
Oryza sativa
Zea mays
Phoenix dactylifera
Petrosavia stellaris
Nymphaea
Liriodendron tulipifera
Cycas taitungensis

nad3

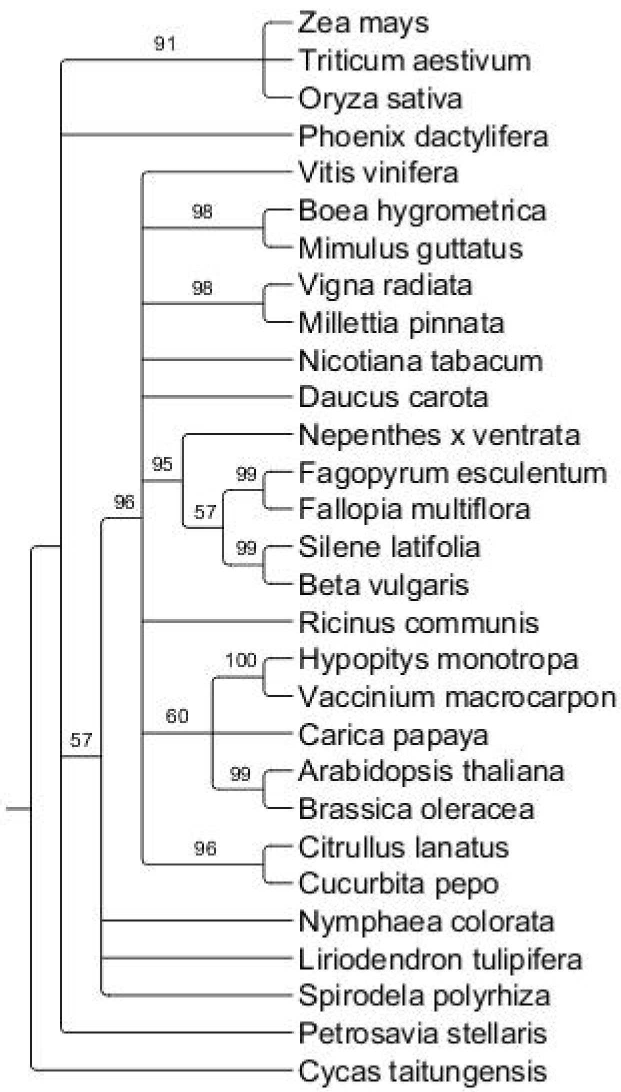
Zea mays
Triticum aestivum
Oryza sativa
Phoenix dactylifera
Vitis vinifera
Boea hygrometrica
Mimulus guttatus
Vigna radiata
Millettia pinnata
Nicotiana tabacum
Daucus carota
Nepenthes x ventrata
Fagopyrum esculentum
Fallopia multiflora
Silene latifolia
Beta vulgaris
Ricinus communis
Hypopitys monotropa
Vaccinium macrocarpon
Carica papaya
Arabidopsis thaliana
Brassica oleracea
Citrullus lanatus
Cucurbita pepo
Nymphaea colorata
Liriodendron tulipifera
Spirodela polyrhiza
Petrosavia stellaris
Cycas taitungensis

nad4

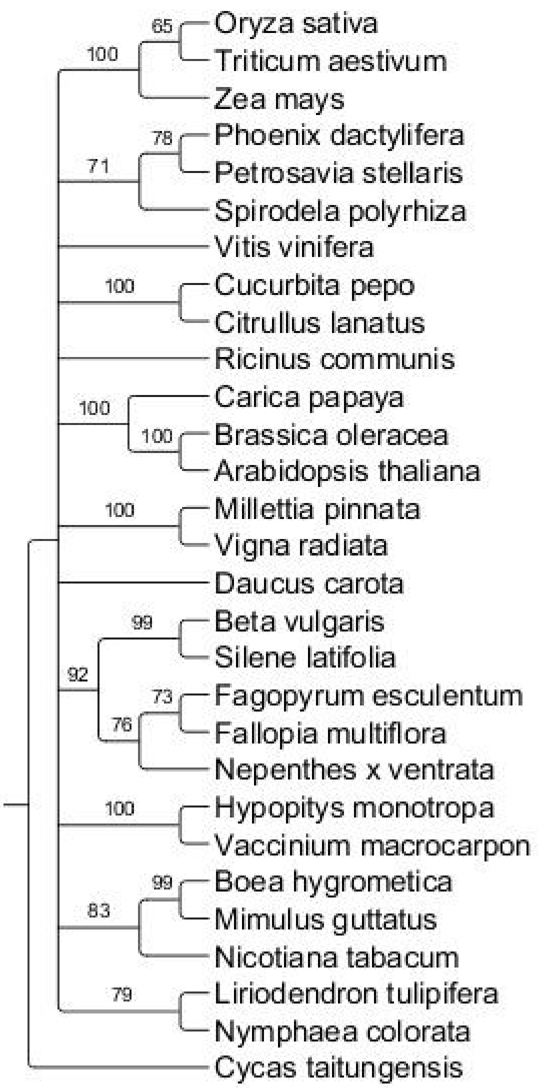

Oryza sativa
Triticum aestivum
Zea mays
Phoenix dactylifera
Petrosavia stellaris
Spirodela polyrhiza
Vitis vinifera
Cucurbita pepo
Citrullus lanatus
Ricinus communis
Carica papaya
Brassica oleracea
Arabidopsis thaliana
Millettia pinnata
Vigna radiata
Daucus carota
Beta vulgaris
Silene latifolia
Fagopyrum esculentum
Fallopia multiflora
Nepenthes x ventrata
Hypopitys monotropa
Vaccinium macrocarpon
Boea hygrometica
Mimulus guttatus
Nicotiana tabacum
Liriodendron tulipifera
Nymphaea colorata
Cycas taitungensis

nad4L

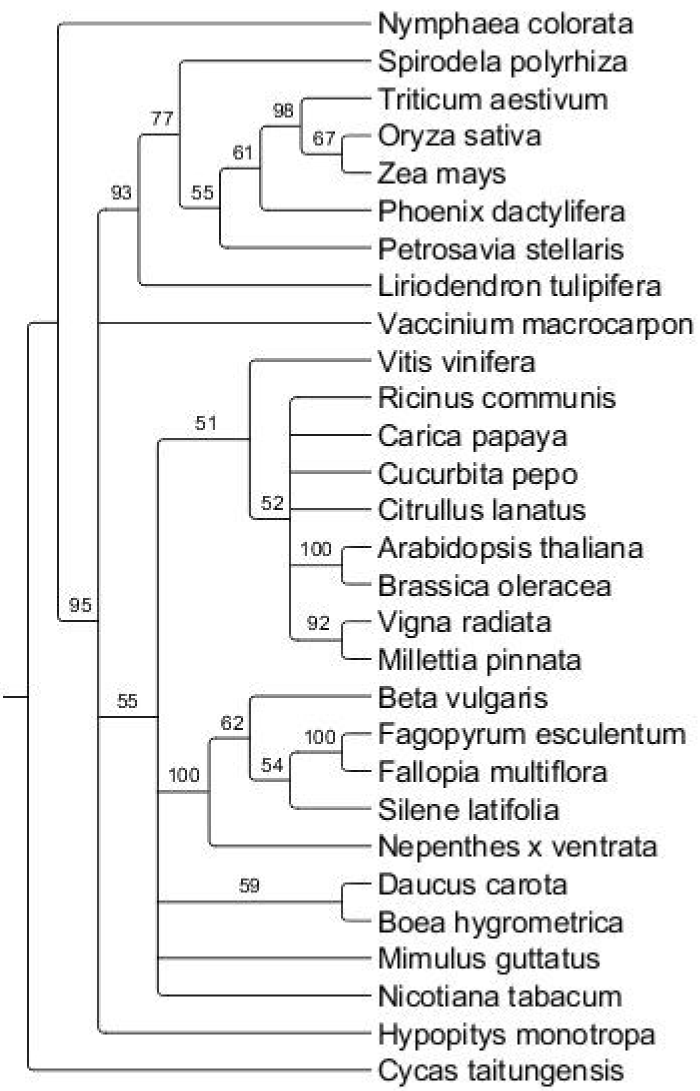

Nymphaea colorata
Spirodela polyrhiza
Triticum aestivum
Oryza sativa
Zea mays
Phoenix dactylifera
Petrosavia stellaris
Liriodendron tulipifera
Vaccinium macrocarpon
Vitis vinifera
Ricinus communis
Carica papaya
Cucurbita pepo
Citrullus lanatus
Arabidopsis thaliana
Brassica oleracea
Vigna radiata
Millettia pinnata
Beta vulgaris
Fagopyrum esculentum
Fallopia multiflora
Silene latifolia
Nepenthes x ventrata
Daucus carota
Boea hygrometrica
Mimulus guttatus
Nicotiana tabacum
Hypopitys monotropa
Cycas taitungensis

nad5

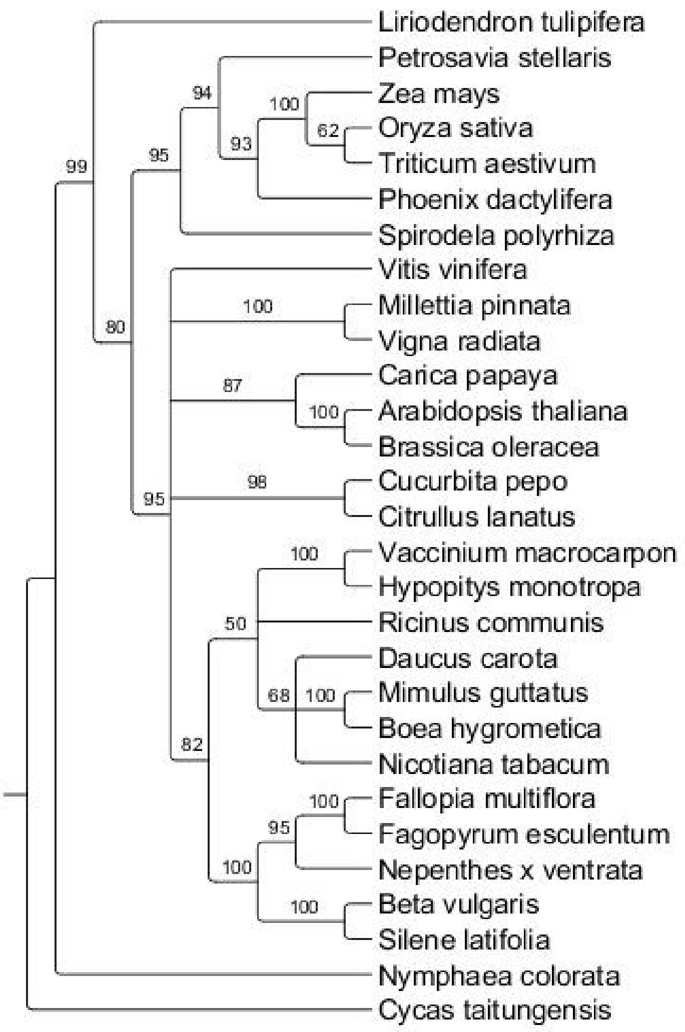
Liriodendron tulipifera
Petrosavia stellaris
Zea mays
Oryza sativa
Triticum aestivum
Phoenix dactylifera
Spirodela polyrhiza
Vitis vinifera
Millettia pinnata
Vigna radiata
Carica papaya
Arabidopsis thaliana
Brassica oleracea
Cucurbita pepo
Citrullus lanatus
Vaccinium macrocarpon
Hypopitys monotropa
Ricinus communis
Daucus carota
Mimulus guttatus
Boea hygrometica
Nicotiana tabacum
Fallopia multiflora
Fagopyrum esculentum
Nepenthes x ventrata
Beta vulgaris
Silene latifolia
Nymphaea colorata
Cycas taitungensis
99
94
100
62
93
95
80
100
87
100
98
95
100
50
68
100
82
100
95
100
100

nad6

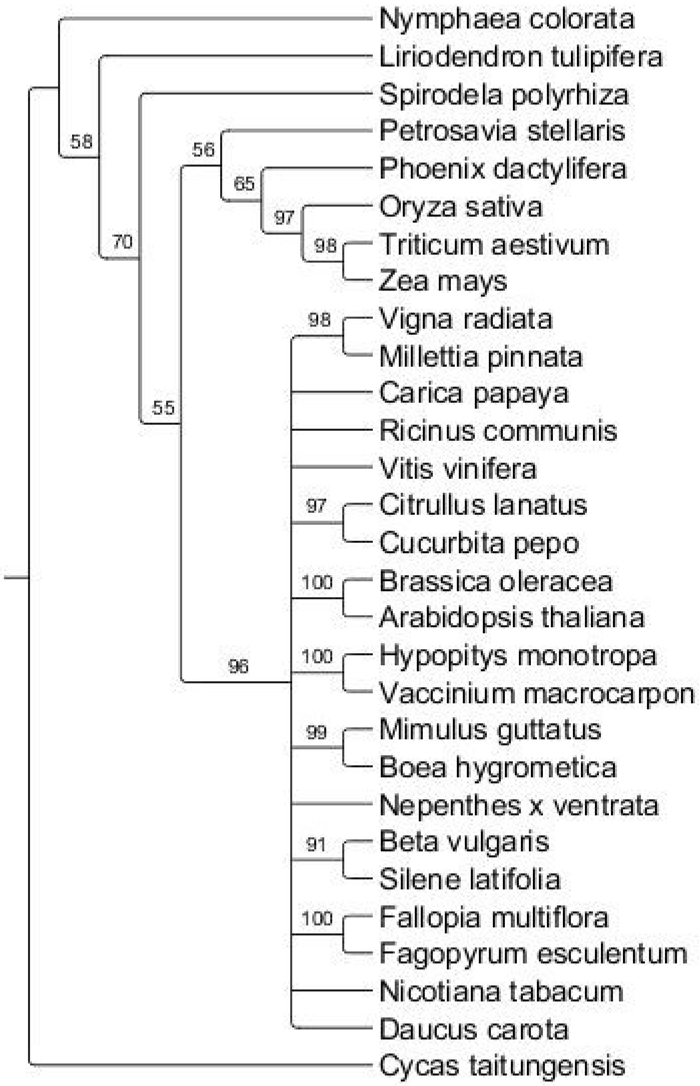
Nymphaea colorata
Liriodendron tulipifera
Spirodela polyrhiza
Petrosavia stellaris
Phoenix dactylifera
Oryza sativa
Triticum aestivum
Zea mays
Vigna radiata
Millettia pinnata
Carica papaya
Ricinus communis
Vitis vinifera
Citrullus lanatus
Cucurbita pepo
Brassica oleracea
Arabidopsis thaliana
Hypopitys monotropa
Vaccinium macrocarpon
Mimulus guttatus
Boea hygrometica
Nepenthes x ventrata
Beta vulgaris
Silene latifolia
Fallopia multiflora
Fagopyrum esculentum
Nicotiana tabacum
Daucus carota
Cycas taitungensis
58
56
65
97
98
70
98
55
97
100
96
100
99
91
100

nad7

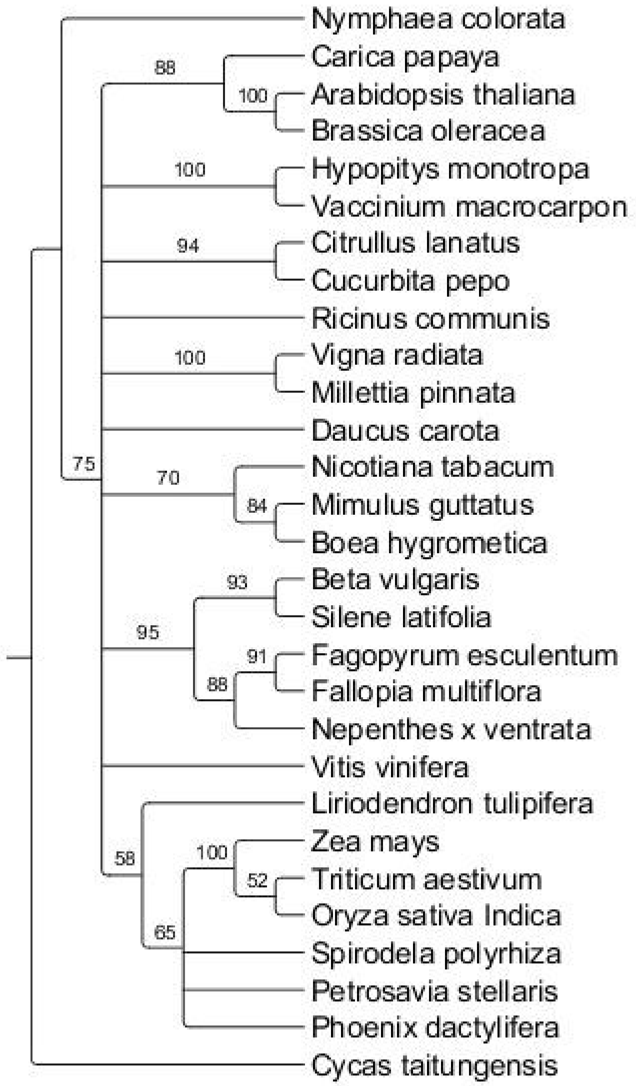

Nymphaea colorata
88
Carica papaya
100
Arabidopsis thaliana
Brassica oleracea
100
Hypopitys monotropa
Vaccinium macrocarpon
94
Citrullus lanatus
Cucurbita pepo
Ricinus communis
100
Vigna radiata
Millettia pinnata
Daucus carota
75
70
Nicotiana tabacum
84
Mimulus guttatus
Boea hygrometica
93
Beta vulgaris
Silene latifolia
95
91
Fagopyrum esculentum
88
Fallopia multiflora
Nepenthes x ventrata
Vitis vinifera
Liriodendron tulipifera
100
Zea mays
58
52
Triticum aestivum
Oryza sativa Indica
65
Spirodela polyrhiza
Petrosavia stellaris
Phoenix dactylifera
Cycas taitungensis

nad9

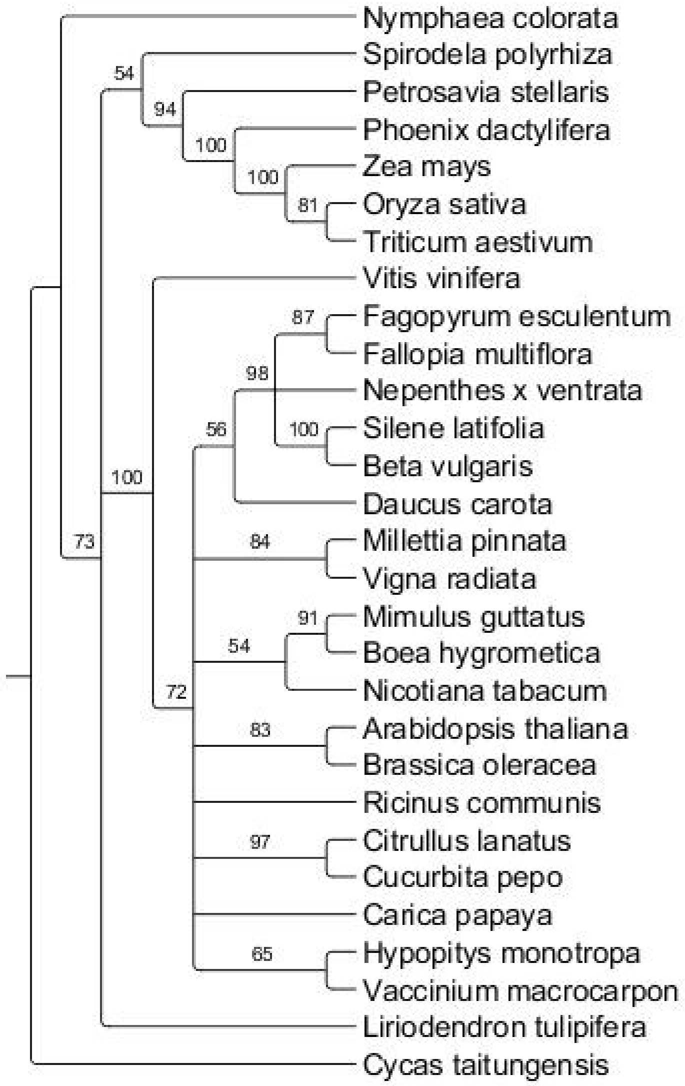

Nymphaea colorata
54
Spirodela polyrhiza
94
Petrosavia stellaris
100
Phoenix dactylifera
100
Zea mays
81
Oryza sativa
Triticum aestivum
Vitis vinifera
87
Fagopyrum esculentum
Fallopia multiflora
98
Nepenthes x ventrata
56
100
Silene latifolia
Beta vulgaris
100
Daucus carota
73
84
Millettia pinnata
Vigna radiata
91
Mimulus guttatus
54
Boea hygrometica
72
Nicotiana tabacum
83
Arabidopsis thaliana
Brassica oleracea
Ricinus communis
97
Citrullus lanatus
Cucurbita pepo
Carica papaya
65
Hypopitys monotropa
Vaccinium macrocarpon
Liriodendron tulipifera
Cycas taitungensis

rps3

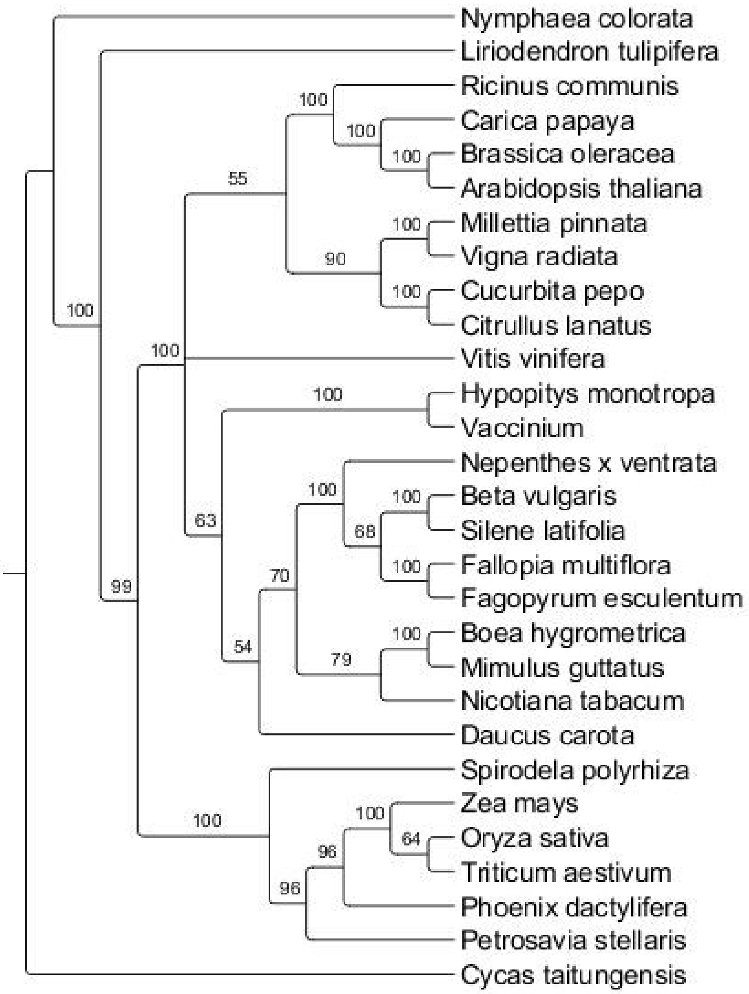
Nymphaea colorata
Liriodendron tulipifera
Ricinus communis
Carica papaya
Brassica oleracea
Arabidopsis thaliana
Millettia pinnata
Vigna radiata
Cucurbita pepo
Citrullus lanatus
Vitis vinifera
Hypopitys monotropa
Vaccinium
Nepenthes x ventrata
Beta vulgaris
Silene latifolia
Fallopia multiflora
Fagopyrum esculentum
Boea hygrometrica
Mimulus guttatus
Nicotiana tabacum
Daucus carota
Spirodela polyrhiza
Zea mays
Oryza sativa
Triticum aestivum
Phoenix dactylifera
Petrosavia stellaris
Cycas taitungensis
100
100
100
55
100
90
100
100
100
100
100
63
100
100
68
100
70
54
100
79
99
100
100
64
96
96

rps4

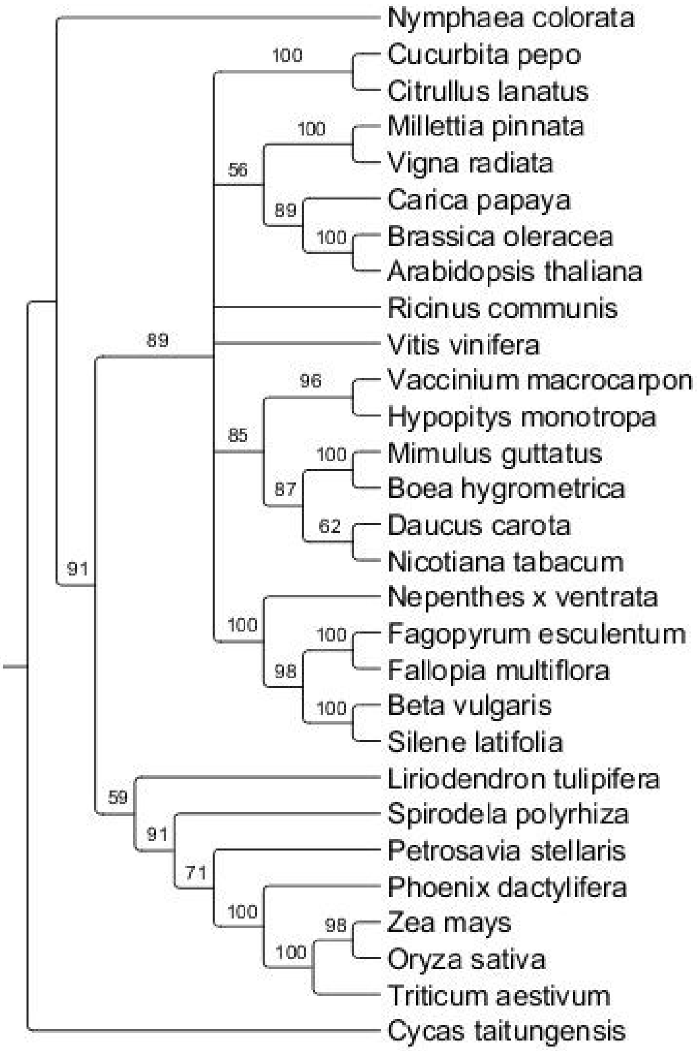
Nymphaea colorata
Cucurbita pepo
Citrullus lanatus
Millettia pinnata
Vigna radiata
Carica papaya
Brassica oleracea
Arabidopsis thaliana
Ricinus communis
Vitis vinifera
Vaccinium macrocarpon
Hypopitys monotropa
Mimulus guttatus
Boea hygrometrica
Daucus carota
Nicotiana tabacum
Nepenthes x ventrata
Fagopyrum esculentum
Fallopia multiflora
Beta vulgaris
Silene latifolia
Liriodendron tulipifera
Spirodela polyrhiza
Petrosavia stellaris
Phoenix dactylifera
Zea mays
Oryza sativa
Triticum aestivum
Cycas taitungensis
100
100
56
89
100
89
96
85
100
87
62
91
100
100
98
100
59
91
71
100
98
100
