# Supplementary figures and images for: Mitochondrial Genome of Fagopyrum esculentum and the Genetic Diversity of Extranuclear Genomes in Buckwheat
Source: Plants (Basel). 2020 May 12;9(5):618. doi: 10.3390/plants9050618 (PMC7285332; doi:10.3390/plants9050618)

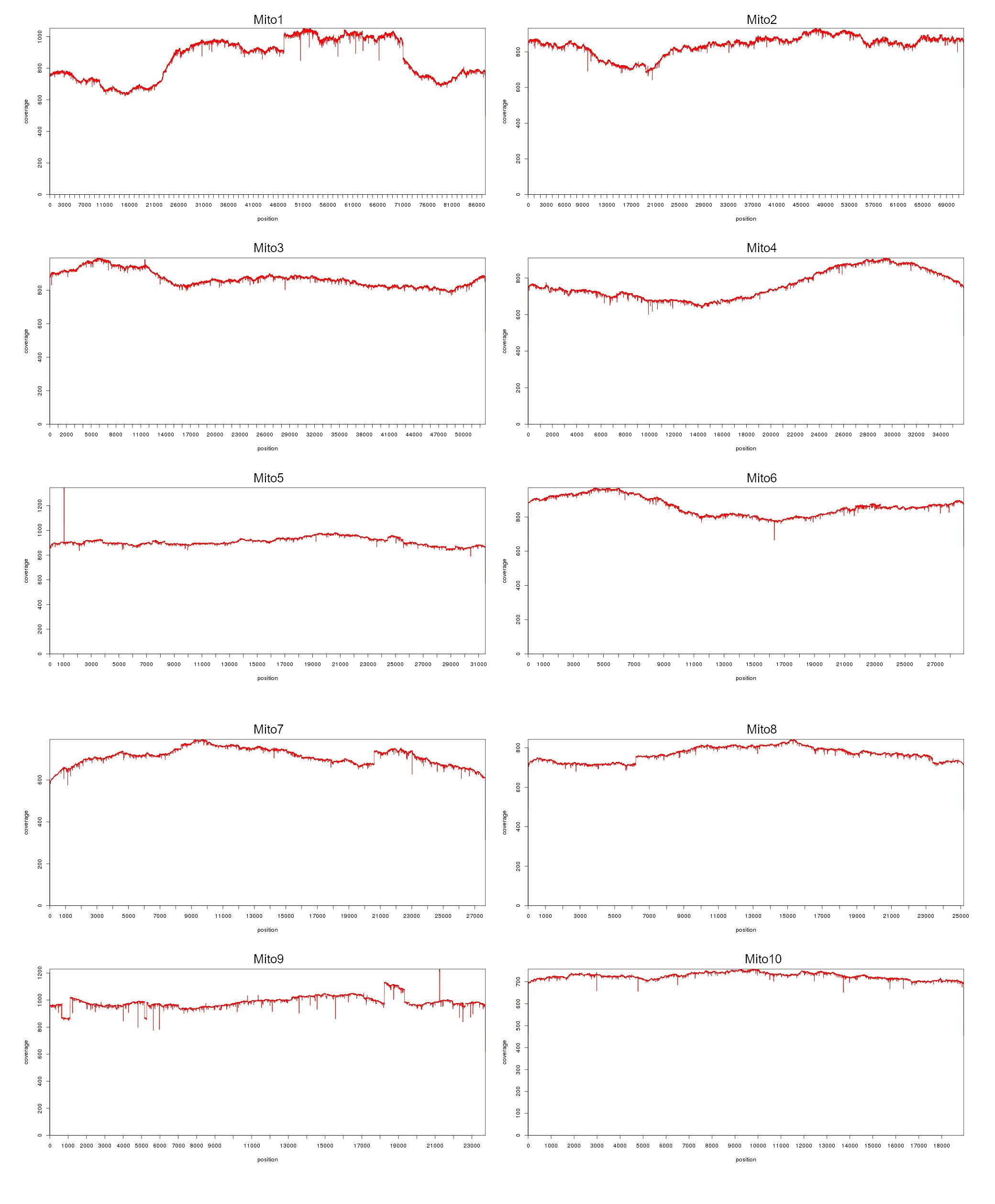

Supplement: Supplementary file 1 [file plants-09-00618-s001.zip › Figure_S1.jpg]
